# Lessons Learnt from Monitoring the Etna Volcano Using an IoT Sensor Network through a Period of Intense Eruptive Activity

**DOI:** 10.3390/s24051577

**Published:** 2024-02-29

**Authors:** Laurent Royer, Luca Terray, Maxime Rubéo-Lisa, Julien Sudre, Pierre-Jean Gauthier, Alexandre Claude, Salvatore Giammanco, Emilio Pecora, Paolo Principato, Vincent Breton

**Affiliations:** 1 Laboratoire de Physique de Clermont Auvergne, CNRS/IN2P3, Université Clermont Auvergne, F-63000 Clermont-Ferrand, France; laurent.royer@clermont.in2p3.fr (L.R.); luca.terray@uca.fr (L.T.); maximerubeo@hotmail.fr (M.R.-L.); julien.sudre@uca.fr (J.S.); alexandre.claude@clermont.in2p3.fr (A.C.); 2 Laboratoire Magmas et Volcans, CNRS/INSU, Université Clermont Auvergne, F-63000 Clermont-Ferrand, France; pierre-jean.gauthier@uca.fr; 3 Osservatorio Etneo, Istituto Nazionale di Geofisica e Vulcanologia, 95125 Catania, Italy; salvatore.giammanco@ingv.it (S.G.); emilio.pecora@ingv.it (E.P.); paolo.principato@ingv.it (P.P.)

**Keywords:** IoT, wireless sensor networks, sensors, LoRaWAN, volcano monitoring

## Abstract

This paper describes the successes and failures after 4 years of continuous operation of a network of sensors, communicating nodes, and gateways deployed on the Etna Volcano in Sicily since 2019, including a period of Etna intense volcanic activity that occurred in 2021 and resulted in over 60 paroxysms. It documents how the installation of gateways at medium altitude allowed for data collection from sensors up to the summit craters. Most of the sensors left on the volcanic edifice during winters and during this period of intense volcanic activity were destroyed, but the whole gateway infrastructure remained fully operational, allowing for a very fruitful new field campaign two years later, in August 2023. Our experience has shown that the best strategy for IoT deployment on very active and/or high-altitude volcanoes like Etna is to permanently install gateways in areas where they are protected both from meteorological and volcanic hazards, that is mainly at the foot of the volcanic edifice, and to deploy temporary sensors and communicating nodes in the more exposed areas during field trips or in the summer season.

## 1. Introduction

As long as 15 years ago, wireless sensor networks were identified as promising tools for the surveillance of active volcanoes [1,2]. On one hand, IoT wireless sensor networks open up the perspective to densely cover volcanic edifices, including the areas most exposed to natural hazards, with cheap and low-consumption sensors providing information in quasi-real time. On the other hand, deploying such a network on active volcanoes involves well-identified challenges [3]. The main ones are the energy supply needed for several months of autonomy, the signal quality between gateways and nodes to collect sensor data, and the access to the internet required for data transfer from the gateway to the analysis end point. In addition, the network components are potentially exposed to extreme conditions related both to local weather (very strong winds, heavy snowfalls, and very low temperatures) and to volcanic activity (acidic plumes, damages due to ash, and volcanic bombs).

All these challenges have in practice considerably limited the adoption of IoT technologies to monitor active volcanoes. The first pioneering works explored the deployment of an IoT network collecting seismic and infrasonic signals [1,4]. Performance evaluations were conducted to identify the optimum number of sensors to be deployed a posteriori based on simulation results, considering throughput, packet loss, and end-to-end delay as metrics to satisfy the real-time requirements [5]. An IoT-based volcano early-warning system was developed and tested to respond to the challenge of monitoring the more than a hundred active volcanoes in Indonesia [6,7]. Its relevance to detect multiple volcanic parameters, including gas emissions (hydrogen sulfide, carbon monoxide), crater temperature, acidity and seismicity was documented [8].

In 2019, a low-power and low-cost wireless network based on LoRa to monitor the soil temperature in thermal anomaly zones in volcanic areas was successfully deployed on Teide volcano in Tenerife (Canary Islands) [9]. In the same period, a network of monitoring stations was installed on Mount Etna, Europe’s most active volcano located in Sicily (Italy), in order to provide a continuous measurement of radon activity in the air [10]. The scientific goal was to test whether radon measurements in the air could be part of the Etna monitoring system. Measuring airborne radon activities requires that sensitive detectors be resistant to hard outdoor conditions, autonomously energetical for several months, relatively compact, and low-cost. To enable the data transfer from the stations to an observatory where the information can be treated and analyzed in real-time, a sensor network infrastructure was deployed on site for data collection and transfer to a data lake located in Clermont Auvergne University data center (Clermont-Ferrand, France) [10]. The choice of LoRaWAN (LoRa Wide-Area Network) was guided by the advantages of private networks compared to public networks in the context of volcano monitoring, mainly the minimal dependence on external actors like private companies and the capacity to tune the network infrastructure to respond to the specific needs of volcanic surveillance [11].

Since its deployment in October 2019, the network has been operational for long periods, although most of the data transmission was interrupted during winter periods due to extreme weather conditions. Following these interruptions of services, the damaged sensors were replaced during field trips in 2020 and 2021.

On 13 December 2020, the Etna volcano entered a new eruptive phase, resulting in an exceptional series of over 60 paroxysmal episodes at the Southeast crater, displaying increased Strombolian activity from the summit craters, with lava fountains feeding several km high eruptive columns and ash plumes, as well as lava flows [12]. Each paroxysmal episode lasted a few hours and was sometimes preceded (but more often followed) by short-lived lava flow output from the crater rim.

This intense eruptive activity lasted for one year and caused major damage to the probes and communicating nodes located close to or on the rim of summit craters. To reduce the potential financial costs of further damage while pursuing the collection of highly valuable scientific data in a period of strong volcanic unrest, the choice was taken to deploy cheaper ground-based thermal measurement devices. The thermal surveillance of volcanoes has been investigated for decades as a tool to predict eruptions [13]. Volcanic activity is always accompanied by the transfer of heat from the Earth’s crust to the atmosphere. This heat can be measured from space [14] or by using ground-based thermal measurements. For example, variations in fumarole temperature, size, number, and location, as well as gas output and chemistry, were shown to be correlated to the eruptive activity of Vulcano (Sicily) [15].

The interest in monitoring fumarole temperatures was also explored at Mount Etna [16]. The time variation in the temperature of a fumarole located in the summit area was recorded from July to September 2008 using two sensors located along a fracture near the rim of the volcano’s central crater. In another study of hydrothermal processes governing the geochemistry of fumaroles fields near summit area, soil temperatures were acquired hourly at depths of 10 and 30 cm by automated stations based on data loggers connected to digital temperature smart sensors [17]. Temperatures were measured every 4 min and their hourly averages were stored in the permanent memory of the logger. This experiment showed that the continuous monitoring of soil temperature in fumaroles located around the craters is feasible, and it could help evaluate the volcano state of activity [17].

During a field trip in September 2021, a new network of resistive temperature detectors (RTDs) was deployed to monitor the volcano activity. This trip was also an opportunity to reorganize the network architecture and improve its robustness in order to better cope with volcanic hazards. A major choice was to move the gateways away from the mountain top into locations where they were completely protected both from volcanic and meteorological hazards. The sensors deployed close to the summit areas were able to transmit data until a major storm destroyed most of them. The period of intense eruptive activity ended in 2022, and the opportunity to further validate the relevance of the new architecture took place in August 2023 during a new field trip focused on analyzing the volcanic plume. The mission took place only a couple of weeks after a paroxysm, during a period when the volcano was again very active.

The present paper reports on our failures and successes over a 4-year-long period. It is to our knowledge the first documented return of experience following the continuous operation of an IoT sensor network on an active volcano for several years, including during a period of intense eruptive unrest. The existing literature documents the development of an IoT-based monitoring system [6,7], feasibility studies and performance evaluation [5], and short-term deployments [1,2,9]. Our documented experience aims at providing useful advice to teams interested in equipping active volcanoes with IoT infrastructures.

The paper is organized as follows: after the introduction, Section 2 describes the material and methods. Section 3 presents the results from September 2021 and August 2023 field trips that are discussed in Section 4. Finally, Section 5 provides a conclusion, and some perspectives are drawn in Section 6.

## 2. Material and Methods

**Figure 1:**
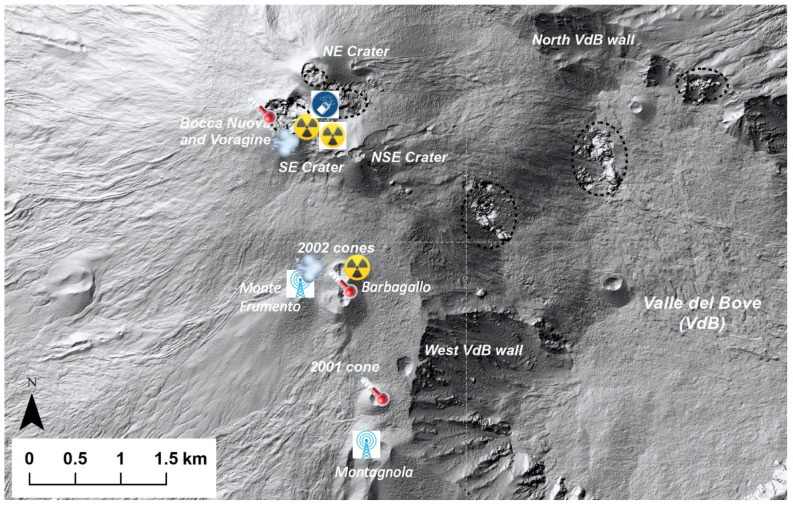
Digital elevation model of Etna summit crater area coming from 2015 Pleiades satellite data (from [18]). Sensor data have been collected since September 2019 using the gateway (blue antenna icon) located at Montagnola. Following the destruction of the radon probes (yellow and black radioactivity icons) deployed in September 2020 on the Bocca Nuova crater rim and in the vicinity of the Southeast crater complex (Barbagallo craters) during volcanic paroxysms, resistive temperature detectors (red icon) were installed in September 2021 for temperature monitoring of fumaroles and thermal anomalies in areas of interest that were less exposed to the eruptive products. After their destruction during the winter season of 2021–2022, no new sensors were deployed until August 2023 when gas detectors (blue circle) were installed alongside an anemometer (cloud symbol) on the Bocca Nuova rim.

This section briefly presents the whole operational chain from the devices deployed on the field to the data lake in Clermont-Ferrand. As shown in Figure 1, sensors were initially installed on Etna summit area in September 2019, and then moved in September 2020 just before a period of intense eruptive activity.

### 2.1. The Sensors

The initial set of sensors deployed in September 2019 were composed of two Algade ÆR+ radon probes that had been tropicalized in order to be operated continuously in harsh volcanic conditions with an autonomy of several months [19]. The tropicalized probe is called ÆR-TT and is available upon request to the Algade company. One radon probe was installed at Monte Frumento along with a meteorological station, while the other radon probe was located close to Torre del Filosofo in the Barbagallo craters (formed during the 2002–2003 eruption) at about 1.8 km distance from the gateway antenna located in the INGV (Istituto Nazionale di Geofisica e Vulcanologia) shelter at Montagnola.

**Figure 2:**
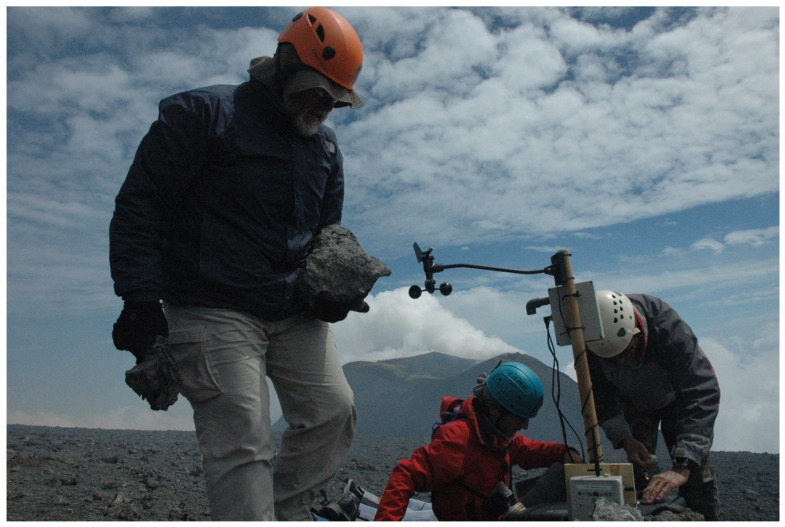
Installation of two probes to measure airborne and soil radon together, with an anemometer, on Bocca Nuova rim in September 2020. Eruptions of the new Southeast crater in the background resulted in the total destruction of these sensors.

The Monte Frumento radon probe was moved in September 2020 to the rim of Bocca Nuova central crater, in an area of significant radon degassing, together with an anemometer [20]. A second radon probe was added to measure radon emissions from the ground on the same spot (Figure 2).

After the destruction of these radon probes together with their communicating nodes upon the intense lava fountaining activity that took place at Southeast crater between late 2020 and summer 2021, a new set of sensors was installed in September 2021 to monitor the volcano. Indeed, in the intense eruptive context characterized by a multiplication of paroxysms and lava flows, a set of sensors was deployed at medium altitude along the south rift zone to continuously monitor well-known heat release areas and possibly detect thermal anomalies related to the ascent of magma prior to eruptive events [21]. For our study, the chosen resistance temperature detectors were Platinum Resistance Pt100 and Pt1000 Class B Sensors with Teflon^®^-insulated lead [22]. These high-quality platinum resistance thermometer probes are manufactured with an aluminum alloy weatherproof connection head and provide a wide operating temperature that ranges from, respectively, −50 °C to +200 °C (PT100) and −100 °C to +450 °C (PT1000). The stations were destroyed after three months of operation in December 2021.

**Figure 3:**
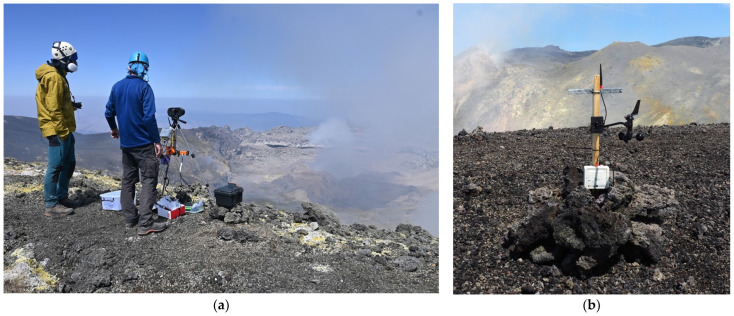
Box of AlphaSense chemical sensors (**a**) and anemometer (**b**) installed together with their communication nodes on Bocca Nuova rim in August 2023.

Finally, a third set of sensors was deployed in August 2023 on the Bocca Nuova crater rim to analyze the chemical composition of the volcanic plume (Figure 3). Indeed, two plastic boxes were designed and constructed using a 3D printer at the LPC laboratory to host four AlphaSense electrochemical sensors. They were connected to communicating nodes, together with an anemometer, in order to measure the concentrations of H_2_S, SO_2_, HCl, and H_2_ in the air, as well as wind direction and speed. SO_2_ and HCl are reliable proxies of the volcanic plume where they are highly concentrated.

### 2.2. The Communicating Nodes

The communicating LoRa end nodes are specific devices, developed within the context of a regional project in Auvergne. They were designed to allow for data reading from a variety of associated sensors, storing them on a memory card and transmitting them at programmable fixed time intervals. As documented in [10], each node has an internal memory to store the data read from a sensor output and is able to send data to the gateway at a programmable time frequency, typically every few minutes. Messages sent by the node are called frames and contain a few measurement results because of the potential for communication failure between the node and the gateway (message collisions, too weak radio signal, weather conditions, …) or between the gateway and the server (backhaul link failure). In order to tackle this problem, the protocol is enhanced with a detection of each communication failure and is programmed to re-send the data later at the next scheduled transmission period.

### 2.3. The Gateways

The reliability of a private LoRaWAN strongly depends on the performances of the gateway, which plays a central role. Choosing gateway locations requires finding a compromise among a number of constraints. The chosen locations should first benefit from a reliable internet connection. Secondly, volcanoes are, by definition, mountainous areas where signal transmission heavily depends on local topography. The gateway locations should be chosen so that the information coming from sensors located in the areas of critical interest for volcanic surveillance is reliably collected. This may require the deployment of multiple gateways with overlapping coverages. Finally, while IoT devices use low-energy-consumption technology that utilizes power generated by solar panels or tiny batteries, commercially available gateway devices require a power source at a similar level as those of ordinary home appliances. Deploying an autonomous gateway on an environmental site without access to electric powerlines and wired internet connections requires installing an energy supply system such as, for instance, solar panels and a connection to either mobile phones or satellite networks. The typical energy consumption of LoRaWAN gateways ranges from 2 to 10 W, requiring at least a 100 W solar installation coupled with a storage capacity above 100 Ah for continuous operation during winter [23]. Beyond the difficulty of installing a solar panel above 2000 m altitude on an active volcano with limited access, such a device is exposed to extreme climatic and volcanic events and so it requires regular maintenance. In addition, its performances are severely impacted during winter by heavy snow falls on Etna.

As a consequence, a first Wirnet IoT station developed by the Kerlink company was installed into a shelter used by the INGV to operate instruments for volcanic surveillance and was located on the Montagnola cinder cone, about 2.5 km south of the summit craters (Figure 1). This building provided energy supply and protection from winter storms, but the LoRa transmission range was reduced because of the RF signal screening induced by the building’s metallic walls. Tests conducted in September 2020 showed that its communication coverage could extend up to the summit craters if the antenna was installed outside the shelter, but this option was not further considered because of the meteorological hazards related to lightning.

**Figure 4:**
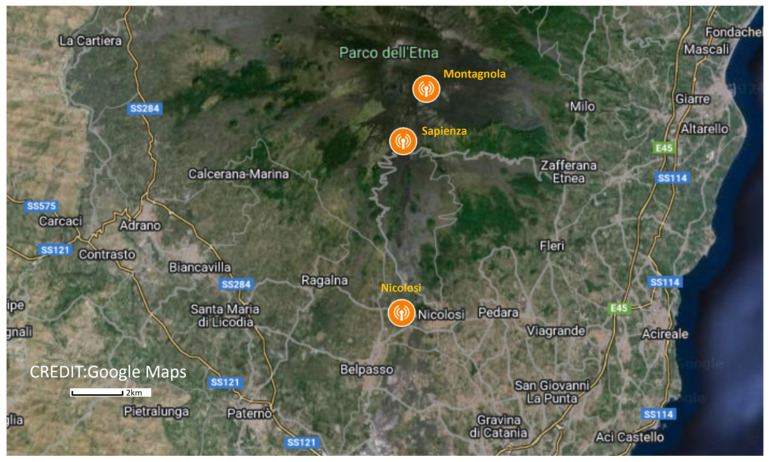
Location of LoRaWAN gateways on Etna. To extend the range of the first gateway, located 2.5 km south of Etna summit craters in a shelter, a second gateway was installed in Nicolosi on the top roof of a school in Nicolosi (10 km south of Etna summit) in September 2021. A third gateway was added at Sapienza touristic area at 1900 m above sea level.

**Figure 5:**
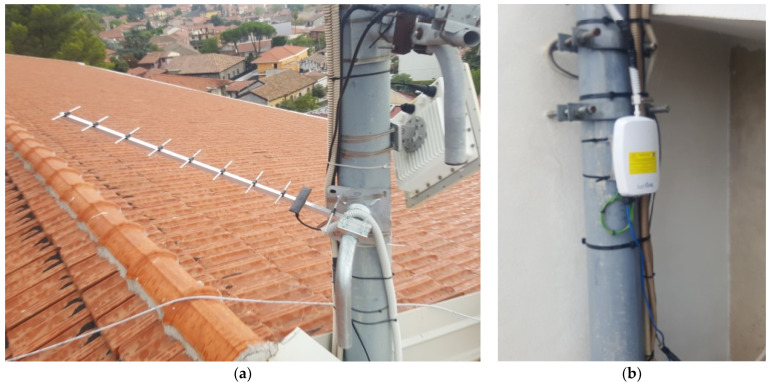
Layout of the gateway set-up on the roof top of Dusmet school in Nicolosi including a Yagi-type antenna pointing toward the Etna summit (**a**) and the Kerlink gateway at the bottom of the same mast connected to the antenna and to the power grid (**b**).

To overcome these limitations, a second gateway was installed at the bottom of the volcanic edifice to fill up the three criteria: mild weather conditions, guaranteed power supply, and reliable access to the internet. This gateway was located in Nicolosi, a village 10 km south of the summit craters at 700 m above sea level (Figure 4). It was equipped with a Yagi-type antenna pointing toward the Etna summit and was installed on the roof top of a school together with other INGNV volcano monitoring devices (InfraRed camera for thermic surveillance) on a mast (Figure 5).

The gateway model is a Kerlink Wirnet iStation with a 3G/4G communication interface and Things Mobile SIM card. Its power is provided by a POE module located inside the INGV electric box.

In addition to those installed in Nicolosi and Montagnola, a third gateway was deployed in the Sapienza touristic area, 1900 m above sea level. In addition to the volcanological interest of the area located in the southern rift zone, where several lateral eruptions have taken place in the past, the motivations for choosing this site were the access to the power line and the interest expressed by the souvenir shop owners to contribute to the collection of scientific data. Its main drawback is the limited coverage due to surrounding terrain.

### 2.4. The Clermont-Ferrand University Data Lake

The data from the Etna sensor network are collected on the Clermont Auvergne University environmental cloud (CEBA, Cloud Environmental au Bénéfice de l’Auvergne) [10]. This data lake was developed to respond to the growing need for reliable access to high-quality agricultural and environmental data in the context of climate change. The Internet of Things (IoT) has become a widely popular technology for the continuous collection of data from ecosystems of interest. Therefore, one of the CEBA’s main features is the ability to collect and manage data from wireless sensor networks without any prerequisites using Elastic Stack [24]. Compared to other platforms, the CEBA fully supports the management of geographic coordinates at every stage of data management. A comprehensive JavaScript Objet Notation (JSON) architecture has been designed to facilitate multi-stage data enrichment. Data from the wireless network are queried and accessed in near-real time, using a distributed JSON-based search engine. The user is able, according to their rights, to access the Grafana dashboards to consult the data in real time. This becomes particularly important during field trips to test the performances of the devices in quasi-real time. Sensor data transmitted to the Clermont-Ferrand university data lake are available after a few seconds on the smartphones of the team members deploying the network on Etna, allowing for rapid bug fixing and performance evaluation.

## 3. Results

### 3.1. Sensor Network Deployed during September 2021 Field Trip

As documented in our previous paper, the first deployment of the sensor network was initiated in October 2019 [10]. In September 2020, two airborne radon probes were relocated on the identified areas of interest on the rim of the Bocca Nuova central crater (Figure 1 and Figure 2), while a third radon probe was kept at its initial location on Mt. Barbagallo (Figure 1). Starting December 2020, a succession of paroxysms at the Southeast crater resulted in the complete destruction of all radon probes and their communicating nodes, either because of lava bombs on the Bocca Nuova rim or because of successive deposits of burning ashes at Mt. Barbagallo. Unfortunately, the only gateway in operation at the time was located inside the Montagnola shelter (see Figure 4) and was unable to communicate with the nodes installed at the Bocca Nuova crater. As a result, the radon and weather stations installed in 2020 at Bocca Nuova remained silent and all the data were lost.

This painful experience led us to rethink the network architecture to better consider the risk of destruction in the case of eruption. Reduced sensor exposure to eruptive hazards was achieved by switching from radon surveillance in plume and soil at the summit areas to the temperature monitoring of fumaroles and thermal anomalies along active faults on the edifice at medium altitude.

Table 1 provides a list of the IoT devices deployed in September 2021, their location, GPS coordinates, and altitude.

Table 2 documents the distance, expressed in kilometers, between the nodes and the gateways. The distance is provided in bold in absence of natural obstacles between the nodes and the gateways.

#### 3.1.1. Performances of the Deployed Network

The choice of the LoRa radio parameter settings has a direct impact on the autonomy of the communicating nodes, but also on the communication range. Moreover, with the LoRaWAN protocol, the payload depends on the data rate (DR) value selected (in our case, 51 bytes with DR = 0 up to 222 bytes with DR = 4 to 7) [10].

Table 3 shows the data loss rates for the communicating nodes that were configured with different payloads (DR = 0 and DR = 5). These rates are compared to the failure rate of the first transmission. When DR = 0, the payload is too small for two measurements to fit into one single frame, and only the last-acquired data can be sent; as a consequence, the total data loss rate is equal to the failure rate of the first transmission. When DR = 5, the measurements can be recovered thanks to the possibility to re-send data when a communication failure is detected. This feature results in less than 3% data loss for node FRA1, while its first transmission failure rate reaches 36%. In the same way, data loss is cancelled for node ESAM thanks to the resending of the data that were not received.

The connection to the Bocca Nuova node (BNAS) was lost after a violent storm on 24 October 2021, resulting in a reduced number of packets expected. Before that day, its data loss rate was only 2%, confirming the efficient data exchange from the summit area to the Nicolosi gateway despite the 15 km distance separating them.

**Figure 6:**
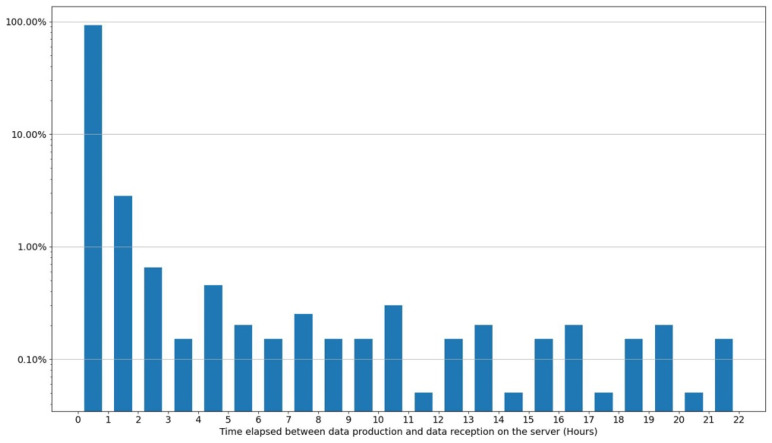
One-dimensional histogram displaying the probability distribution for the time elapsed between measurements and their transmission by the ESAM node. The large majority (>90%) of the data frames are successfully delivered to the gateway at first attempt (first histogram bin), but a small fraction fails, resulting in resubmission and delayed reception of the data (all other bins).

Figure 6 displays, on a logarithmic scale, the elapsed time between the measurement and its transmission at DR = 5 by the ESAM node that was configured to transmit data once an hour. The figure confirms that more than 90% of the data are transmitted within one hour, and therefore at the first attempt. It also shows that data have been transmitted up to 20 h after their production. Because of the short distance—a few meters—between the ESAM node and the gateway installed at Sapienza, the 7% failure rate of the first transmission cannot be explained by a weak LoRa signal. The transmission loss is more probably due to some interruptions in the internet access through the site’s 4G coverage. The use of Wi-Fi or a wired internet connection as available at this location could significantly improve the robustness of the backhaul link.

#### 3.1.2. Communication Sharing between Gateways

**Figure 7:**
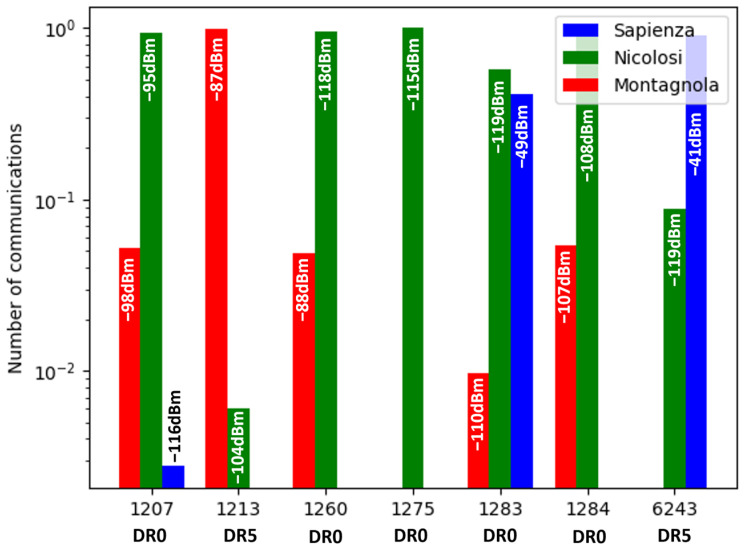
Communication sharing between the 7 nodes and the 3 gateways. For each node, the normalized communication rate with Montagnola (red), Nicolosi (green), and Sapienza (blue) is provided, together with the average signal strengths expressed in dBm.

When a node initiates a communication with the network server, the latter selects the gateway with the best SNR to carry on the data transmission. Figure 7 documents the sharing of communications for each node between the three gateways as well as the average signal strength expressed in dBm. For instance, node SCO2-1207, located south of the Barbagallo crater at 2900 m above sea level, was only 1400 m away from the Montagnola gateway but communicated about 95% of its data to the Nicolosi gateway located more than 13 km away. The average signal strengths are comparable: −98 dBm for Montagnola and −95 dBm for Nicolosi. Node MFRU-1284, located only 450 m from node SCO2-1207, shows a similar pattern regarding its preferred communication with the Nicolosi gateway.

More surprisingly, frames emitted by node SCO1-1260 also transit mainly through Nicolosi at 12 km, despite the node’s location being only 300 m from the Montagnola gateway. The Nicolosi gateway is selected by the server although the Montagnola gateway signal strength is on average 30 dBm greater. A possible explanation is the difference in the quality of the mobile networks available at the gateway locations. Latency at the Nicolosi primary school benefits from the proximity of a dense urban network. Shorter latency times to reach the server from Nicolosi could explain why this route is preferred for data transfer.

The Sapienza gateway is clearly the gateway least used by the communicating nodes. As stressed earlier, the motivations for installing a gateway at Sapienza touristic area were much more related to the access to the power grid and the availability of volunteers for instrument maintenance, whereas its location results in a limited coverage of the regions of interest close to the summit area. The two nodes ESAP-1283 and ESAM-6243 located a few meters away used the gateway for data transfer with excellent signal strength. Surprisingly, a large fraction of the frames transited through Nicolosi despite the much reduced signal strength. Once again, the difference in the quality of the mobile networks between Nicolosi and Sapienza could explain these results.

Node FRA1-1213 is the node with the least exchanges with the Nicolosi gateway. The transmission of its signal is strongly attenuated by the mountain relief, with this node being on the bottom of the northern flank of the Montagnola cone (Figure 1). Last but not least, communication from the Bocca Nuova summit crater node BNAS-1275 occurred exclusively through the Nicolosi gateway 15 km away, provided that the highest spreading factor (DR = 0) was chosen for the LoRa modulation.

#### 3.1.3. Temperature Measurements

Temperature measurements were collected from 16 September to 16 December 2021, when two paroxysmal episodes occurred on 21 September and 23 October.

Starting with the node located on the Bocca Nuova summit crater, winter storms damaged the communicating nodes one after the other, while resistance temperature detectors underground were safely recovered during a maintenance mission at the end of December 2021. This underlines that the weak point of the stations is the part above ground level, exposed to severe weather events.

**Figure 8:**
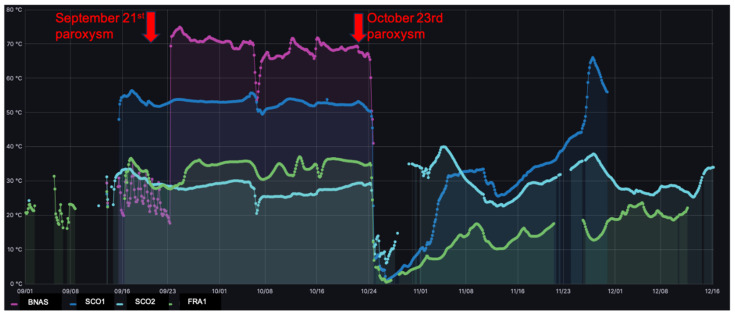
Temperature data as a function of time (UTC) at 50 cm below ground at the 4 sites monitored (BNAS in purple = Bocca Nuova central crater rim, SC01 in blue = 2001 ash cone, SC02 in green = 2002 ash cone, FRA1 in pastel blue = flank of 2002 ash cone) during September–December 2021 monitoring period. Red arrows correspond to the paroxysmal events of 21 September and 23 October 2021.

Figure 8, extracted from the Etna monitoring dashboard at Mesocentre Clermont Auvergne, displays the temperature data collected at 50 cm underground at the different points of interest (BNAS = Bocca Nuova central crater rim, SC01 = 2001 ash cone, SC02 = 2002 ash cone, FRA1 = flank of 2002 ash cone). The gaps in the temperature curves correspond to the packets lost due to transmission failures. These curves display complex patterns with upper values above 70 °C and lower values down to 0 °C.

**Figure 9:**
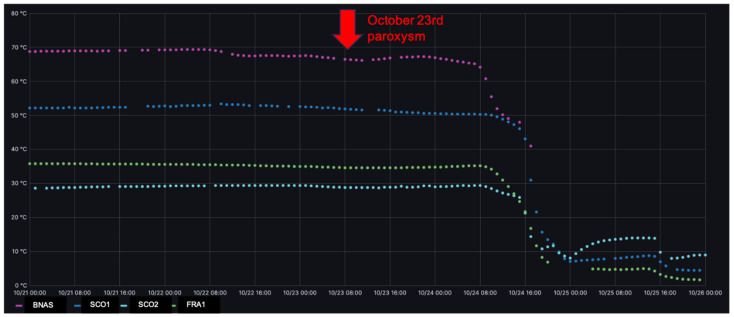
Temperature data as a function of time (UTC) at 50 cm below ground at the 4 sites monitored (BNAS in purple = Bocca Nuova central crater rim, SC01 in blue = 2001 ash cone, SC02 in green = 2002 ash cone, FRA1 in pastel blue = flank of 2002 ash cone) for the 3 days before and the 3 days after the paroxysmal event of 23 October 2021. The red arrow position and width provide the eruption time and duration.

A simultaneous steep decrease in all temperature measurements was observed on 24 October between 12:00 and 20:00 PM UTC + 1 time, just 24 h after a paroxysmal event. Its interpretation must also take into account the extremely heavy rains that hit the eastern part of Sicily that same day, resulting in heavy snowfalls at high altitude. To confirm the meteorological origin of the observed temperature drop, Figure 9 displays a comparison of the temperature profiles in the 3 days before and the 3 days after the paroxysmal events that occurred on 23 October.

As can be seen in Figure 9, no significant change in any of the four temperature recordings was observed before the eruptions or in the following 24 h. Significant changes were instead observed later on, but their interpretation requires further investigation that is beyond the scope of this paper.

Of high interest from an IoT perspective, it should be noted that during the first paroxysm on 21 September, communication with the nodes deployed on the volcanic edifice was not lost even during the lava fountain from 8 to 9:30 a.m. UTC. Similarly, communication was conserved during the second paroxysm on 23 October during the lava fountain from 8:30 to 10:30 a.m. UTC.

Communication with the sensors deployed on the field was lost during winter 2021–2022 due either to meteorological conditions or volcanic eruptions. The only two sensors that survived were those installed in the courtyard of the Esagonal souvenir shop in the Sapienza touristic area (nodes ESAP-1283 and ESAM-6243), a few meters away from the Sapienza gateway.

### 3.2. Results during August 2023 Field Trip

Following the destruction of all the sensors on the volcanic edifice, except for those located in the Sapienza touristic area, in August 2023, a new strategy was adopted to take advantage of the Etna gateway infrastructure while limiting the destruction of instruments. Instead of bringing a new set of sensors to leave on the edifice, the sensors were deployed in the most relevant area for the mission of our scientific program, namely on the rim of the Bocca Nuova summit crater at 3235 m above sea level. Their location was changed during the field trip, and they were dismantled at its end. All nodes were configured with the same payload using DR = 5. This offered the possibility to re-send data when a communication failure was detected.

Table 4 documents the positions of the communicating nodes at different time periods, whereas Figure 3 shows their installation on the crater rim. Connectivity to the gateways was unsuccessful when nodes 1288 and 1302 were set on the crater ground surface. A slightly improved connectivity was achieved when these nodes were installed one meter above ground level (Figure 3a).

Table 5 documents the number of packets received from the nodes by the Nicolosi and Montagnola gateways together with the signal strength. It should be noted that the Sapienza gateway was also operational, but it did not receive any packet from the nodes on the Bocca Nuova rim. The signal strength was comparable to the one measured at node 1275 in September 2021 (see Figure 7), which was also installed on the Bocca Nuova rim. Communication with nodes 1223 and 1271, located at the same place, and with node 1280 was very successful, with about 1300 packets and 511 packets received, respectively. On the other hand, communication with nodes 1288 and 1302 was extremely limited, with only 73 and 0 packets received, respectively. The connectivity was, therefore, very sensitive to each node’s precise location on the crater rim.

Table 6 documents the data loss rates over the same period, which were again very small for nodes 1223, 1271, and 1280, but very high for node 1288 because of its location on the inner side of the crater rim with poor signal strength. For the same reason, node 1302 failed to communicate.

**Figure 10:**
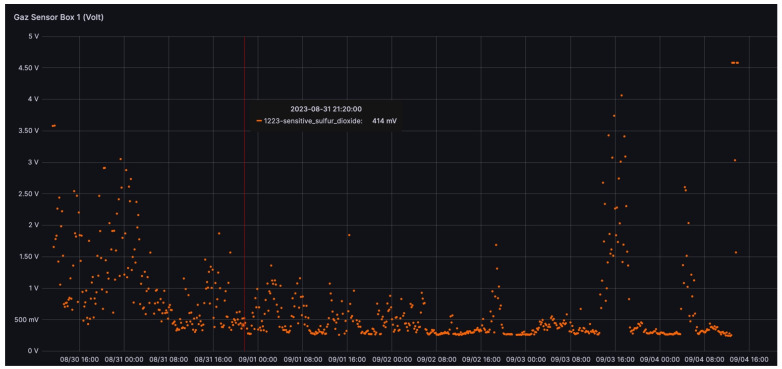
Signal measured in the sulfur dioxide gas sensor connected to the node 1223 during its period of operation on the crater rim. Information on one measurement point is given as an example.

Figure 10 shows the signal measured in the sulfur dioxide gas sensor connected to node 1223 during its period of operation on the crater rim. This signal provides direct information about the presence and density of the volcanic plume. The data collected are still under analysis.

## 4. Discussion

The results presented in the previous chapter document the successes and failures of our attempts to deploy wireless sensor networks on the Etna volcano.

The first lesson is that meteorological conditions require dedicated tropicalized instruments. All the communicating nodes located above 2800 m were destroyed because they had to be installed above the ground surface and so were quickly exposed both to heavy snow falls and to extremely cold temperatures. The only sensors that were not destroyed during the winter following their deployment on the volcanic edifice were a pluviometer and a weather station. They were actually installed in September 2021, together with a gateway, in the external courtyard of a souvenir shop in the Sapienza touristic area at 1900 m altitude. Without requiring any maintenance, they have continuously produced and transmitted data for more than 2 years.

On a more positive note, the choice to install gateways in locations where they were protected from extreme weather has proven to be a real success. However, the consequence of this approach is the need to install multiple gateways either in protected shelters at high altitude, where their coverage is however limited by local geography, or at low altitude around the volcano. Even in the case of a very large volcano like Mt. Etna, our results show that a 15 km distance does not prevent continuous data collection on summit areas. However, the transmission quality depends very much on the precise location and set-up of the communicating nodes. Special care must be taken to install nodes above the ground and to point their antenna in the direction of the targeted gateway. Having quasi-real time access to the data on the CEBA cloud is very important in order to test the network performances on the field.

Full coverage of the Etna summit craters will require the installation of gateways on the eastern, northern, and western sides of the edifice. Within 15 km from the Etna summit, many towns could easily be equipped with gateways.

## 5. Conclusions

This paper is a follow-up on a previous paper describing an IoT network of radon probes on the Etna volcano in Sicily. It documents, for the first time, the experience of return following the continuous operation of a network of sensors and gateways using a low-power wide-area transmission technology on an active volcano for several years. During that period, the sensor network was exposed to over 60 paroxysmic eruptive events.

The first key finding is that extreme winter meteorological conditions have proven to be too harsh for the sensors (temperature and radon air probes) and the communicating nodes that were left on the volcanic edifice. As a consequence, a new strategy was adopted to move the gateways toward the periphery of the volcano to reduce their exposure to bad weather and eruptions. This choice turned out to be extremely successful. All gateways were found to be fully operational 2 years after their installation, without any need for maintenance. Their range is also sufficient to transmit data from the nodes all the way to the crater rims.

The second key finding is that the right strategy for instrumenting active volcanoes in the long term is to install gateways in sheltered DC-powered locations around the edifice at low–medium altitude in order to protect them from extreme weather. The locations should be chosen in such a way that they allow for a good coverage of the areas of interest, but also for high-quality access to internet. Our experience shows that signal transmission works up to 15 km away from the crater rims on the Mt. Etna summit. Using this gateway infrastructure, research teams can connect on demand with sensors and communicating nodes, gaining access to continuous and quasi-real time data collection. Sensors and nodes can be removed at the end of field trips to avoid their destruction later on. This modus operandi was adopted during our 2023 field trip with great success, yielding the most volcanologically relevant data from our IoT deployment.

The third key finding is that the transmission between the sensors, communicating nodes, and gateways was operational during the paroxysmic events that occurred during our final mission trip. This confirms the robustness of private LoraWANs and their relevance for volcanic surveillance.

The limitations of this study include the reduced amount of data that we are able to collect during winter seasons and the long break between our field trips from September 2021 to August 2023. Another limitation is the small coverage of the summit area that was achieved using the gateway located in Nicolosi. It was sufficient for our needs, but it should be enlarged to really have an impact on broader scientific research activities. Another limitation is that our study is focused on Etna, a 1200 km^2^, 150 km circumference, densely populated volcano culminating at 3357 m. If lower-altitude volcanoes are less exposed to meteorological hazards, volcanic hazards are still significant and keeping gateways away from them is a safe strategy. Not all active volcanoes are surrounded by villages as Etna is, so finding sheltered DC-powered locations may be more difficult. However, it should be pointed out that the most important volcanoes to monitor are those in densely populated areas, where such locations should be easier to find.

## 6. Perspectives

The results shown here open up very exciting perspectives. Indeed, thanks to the gateway infrastructure currently operational at Mt. Etna, any team coming with LoRa communicating nodes can plug them to this local network and have quasi-real time data collection and visualization. The current transmission range covers the south flank of the volcano up to the central crater rim, but, as a next step, additional gateways should be installed around the volcano to extend the coverage to other scientifically important sectors of the summit area.

Moreover, the same strategy could be applied to many other active volcanoes that require remote surveillance. The main challenge is to identify the right spots to install the gateways at the bottom of the volcanic edifice to achieve the coverage needed for the different scientific programs. Our gateway is installed on the roof of a public school in Nicolosi where access to the power line and to the internet are excellent. Installing additional gateways in towns and villages around the Etna volcano is easy, the equipment and operational costs are limited (a few hundred euros of equipment and a few euros of internet fee per gateway), and the maintenance is very light.

To monitor the volcano during the winter season, two options can be considered: one is to deploy rugged sensors and communicating nodes specifically designed for resistance to extreme weather and/or acidity and/or temperature, but this comes with a significant additional cost compared to more standard devices and without any guarantee that they will be able to resist over extended time periods and extreme events such as paroxysms. Another approach is to consider the deployment of low-cost sensors. They have received a lot of attention in recent years in various fields [25]. Their reduced resistance and life expectancy can be compensated by deploying multiple devices for the sake of redundancy.

## Figures and Tables

**Table 1:** List of IoT devices deployed, their location, GPS coordinates, and altitude.

Device Name	Device Type	Location	Latitude N	Longitude E	Altitude (m)
NIC	Gateway	Scuola Dusmet, Nicolosi	37°36′50.40″	15°01′08.76″	731
MON	Gateway	INGV shelter, Montagnola	37°43′08.55″	15°0′13.21″	2600
SAP	Gateway	Esagonal, Sapienza area	37°42′0.00″	14°59′57.49″	1903
MFRU	Communicating node n°1284	Monte Frumento, meteorological station	37°43′57.27″	14°59′43.35″	2827
SCO2/node RLT	Communicating node n°1207	Scoria cone 2002, thermal monitoring station	37°43′54.16″	15°0′0.59″	2911
FRA1/Node OPGC	Communicating node n°1213	Scoria cone 2001, thermal monitoring station	37°43′12.90″	15°0′18.30″	2640
SCO1/2001 top	Communicating node n°1260	Scoria cone 2001, thermal monitoring station	37°43′18.52″	15°0′16.84″	2696
ESAP	Communicating node n°1283	Esagonal, pluviometer	37°42′0.00″	14°59′57.49″	1903
ESAM	Communicating node n°6243	Esagonal, meteorological station	37°42′0.00″	14°59′57.49″	1903
BNAS/Bocca Nuova	Communicating node n°1275	Bocca Nuova south, thermal monitoring station	37°44′56.90″	14°59′29.83″	3235

**Table 2:** Distance expressed in kilometers between the nodes and the gateways. The distance is provided in bold in absence of natural obstacles between the nodes and the gateways.

Gateway/Node	MFRU	SCO2	FRA1	SCO1	ESAM	ESAP	BNAS
NIC	**13.48**	**13.35**	12.01	**12.19**	**9.77**	**9.77**	**15.39**
MON	**1.69**	**1.47**	**0.19**	**0.33**	2.26	2.26	**3.56**
SAP	3.75	3.66	2.42	2.59	**0.00**	**0.00**	5.65

**Table 3:** Data loss rate of the communicating nodes configured with DR = 0 (minimum pay load) and DR = 5.

Communicating Node Name	Node Number	Node Location	Data Rate	First Transmission Failure Rate (%)	Number of Packets Received	Number of Packets Expected	Data Loss Rate (%)
SCO2	1207	Scoria cone 2002	0	15%	1663	1968	15%
SCO1	1260	Scoria cone 2001	0	6%	1710	1810	6%
BNAS	1275	Bocca Nuova sud	0	2%	877	937	2%
ESAP	1283	Esagonal, Sapienza	0	46%	1049	1934	46%
MFRU	1284	Monte Frumento	0	12%	1715	1940	12%
ESAM	6243	Esagonal, Sapienza	5	7%	1976	1976	0%
FRA1	1213	Scoria cone 2001	5	36%	1883	1946	3%

**Table 4:** GPS positions, associated sensors, and gateway connectivity of the communicating nodes at different time periods during August 2023 field trip.

Nodes	Sensor Type	GPS Coordinates	Installation Time	Dismantling Time	Connectivity to Gateways	Position
1223	Gas	37.732576° N, 14.995165° E	26 August 2023 14 h 30 min	27 August 2023 16 h	Yes	1 m above ground
1271	Gas	37.732576° N, 14.995165° E	26 August 2023 14 h 30 min	27 August 2023 16 h	Yes	1 m above ground
1288	Gas	37.733392° N 14.997995° E	26 August 2023 11 h 20 min	26 August 2023 16 h	No	On the ground
1302	Gas	37.733392° N 14.997995° E	26 August 2023 11 h 20 min	26 August 2023 16 h	No	On the ground
1223	Gas	37.749065° N, 14.995015° E	30 August 2023	4 September 2023	Yes	1 m above ground
1271	Gas	37.749065° N, 14.995015° E	30 August 2023	4 September 2023	Yes	1 m above ground
1288	Gas	37.748882° N 14.994205° E	30 August 2023	4 September 2023	Yes	1 m above ground
1302	Gas	37.748882° N 14.994205° E	30 August 2023	4 September 2023	No	1 m above ground
1280	Anemometer	37.748718° N, 14.992689° E	29 August 2023	4 September 2023	Yes	1 m above ground

**Table 5:** Communication rate and average signal strength for the 2 gateways used to transmit packets in August 2023 from the nodes on Bocca Nuova rim.

Nodes	Data Rate (DR)	Number of Packets Received	Nicolosi Gateway		Montagnola Gateway	
			Communication Rate (%)	RSSI (dBm)	Communication Rate (%)	RSSI (dBm)
1223	5	1319	100	−114	0	-
1271	5	1259	96	−111	4	−109
1288	5	73	99	−119	1	−110
1280	5	511	90	−108	10	−109
1302	5	0	-	-	-	-

**Table 6:** Quality of data transmission during August 2023 field trip.

Nodes	Number of Expected Packets	Number of Missing Packets	Data Loss Rate
1223	1473	154	10.5%
1271	1342	83	6%
1288	1481	1408	95.1%
1280	519	8	1.5%

## Data Availability

Data are contained within the article.
